# The impact of economic policy uncertainty on corporate social responsibility: A new evidence from food industry in China

**DOI:** 10.1371/journal.pone.0269165

**Published:** 2022-06-10

**Authors:** Fang Su, Nini Song, Haiyang Shang, Shah Fahad

**Affiliations:** 1 School of Economics and Management, Northwest University, Xi’an, China; 2 School of Economics and Management, Shaanxi University of Science & Technology, Xi’an, China; 3 School of Management and Business, Northwest University of Political Science and Law, Xi’an, China; 4 School of Economics and Management, Leshan Normal University, Leshan, China; University of Almería, SPAIN

## Abstract

The ability of the food industry to practice a high degree of corporate social responsibility is related to whether national food safety can be effectively guaranteed. By taking the food enterprises in China’s A-share listed companies from 2009–2018, this paper probes into the influence of China’s macroeconomic policy uncertainty on corporate social responsibility, and depicts the differencent impact of enterprise’s practice of social responsibility under government association and market association, respectively. The results firstly show that, the uncertainty of economic policy has a negative effect on the social responsibility of food enterprises, with a regression coefficient of -0.013. Secondly, nonstate-owned enterprises are more vulnerable to macroeconomic fluctuations in the practice of social responsibility. Thirdly, in the case of greater economic policy uncertainty, enterprises with stronger market connections in the food industry tend to be more conservative in their CSR. The above research results not only verify the path of transmission of economic policy uncertainty to corporate social responsibility, but also provide important ideas and references for improving the level of corporate social responsibility in the food industry and ensuring food safety.

## 1 Introduction

Corporate social responsibility (CSR) has always been the focus of attention from all sections of society [1]. The CSR behavior will have a direct impact on the sustainable development of the company [2]. According to the definition of European Communities, corporate social responsibility refers to companies taking responsibility for their impact on society [3]. It is a concept whereby enterprises integrate social and environmental concerns into their mainstream business operations on a voluntary basis [4]. Corporate social responsibility drives the enterprise to change into the traditional concept of taking economic interests as the only goal, emphasizes the importance of human value in the production link, and emphasizes the contribution of enterprises to consumers, the environment, and society [5]. According to *Research Report on Corporate Social Responsibility of China (2019)* [6], the social responsibility development index of the top 300 Chinese enterprises in 2019 was 32.7 points and about the enterprise development index of more than 50% was below 20 points, which is in the initial stage.

Throughout all industries, the food industry has a naturally inseparable connection with social responsibility [7]. As the food industry is a typical industry of corporate responsibility sensitivity, whether food enterprises can practice corporate social responsibility is related to national food safety. So, there is a need to be alert to the uncertain safety risks that include the lack of corporate social responsibility, especially for companies that manufacture or handle food. To fundamentally guarantee national food safety, it is urgent that food manufacturing companies strengthen the integrity construction and actively practice corporate social responsibility.

In past theoretical exploration and social practice, it has been seen that government, as a social public administration, has become the driver of corporate social responsibility [8]. And the government adopts economic policies to promote and encourage enterprises to act in a way that practices responsibility and sustainable development [9]. In this sense, governments have established a new type of political relationship with companies and stakeholders in civil society, to promote socially responsible and sustainable business practices [10]. There is widespread uncertainty in economic activities [11], and how economic policy uncertainty(EPU) affects macroeconomic activity has been widely analyzed in the theoretical research [12]. For example, some scholars subdivide economic policy uncertainty into fiscal policy uncertainty, monetary policy uncertainty, and trade policy uncertainty [13]. Then some scholars focus on the analysis of the macroeconomic control effect of the monetary policy uncertainty on the economic growth [14] and price stability [15], based on the theory of rational expectations and New-Keynesian economics. In addition, the economic policy uncertainty under China’s supply-side structural reform is gradually increasing [16], and the economic policy uncertainties are also bound to have a certain impact on the industry [17]. Therefore, it is of great significance to comprehensively analyze the impact of economic policy uncertainty on the corporate social responsibility of food industry.

Based on the above mentioned, this paper takes the A-share listed companies in China’s food industry as an example, and explores the impact of economic policy uncertainty on corporate social responsibility. Further, under the corporate heterogeneity of the property rights association and the market association, this paper analyzes the differential impact of economic policy uncertainty on corporate social responsibility. Through the theoretical analysis and empirical research, this paper proposes specific measures for enterprises to better practice their social responsibility. Moreover, this study provides policy reference suggestions for protect national food safety and implement food safety strategies. The contribution of this study is that, first, this paper clarifies the transmission path of economic policy uncertainty with corporate social responsibility. Further, this paper conducts empirical analysis to investigate the influence of China’s economic policy uncertainty on corporate social responsibility. Finally, this paper measures how macro-external uncertainty factors affect the decision-making behavior of food companies in terms of social responsibility.

The remainder of this paper is organized as follows. Section 2 presents the theoretical background and develops the hypotheses; Section 3 describes the methods and data sources; Section 4 reports the empirical results; Section 5 presents related discussion; the last section concludes the paper and offers suggestions and directions for future research.

## 2 Theoretical analysis and research hypothesis

Uncertainty affects economic activities mainly through four endogenous transmission channels; including the cautious savings effect channel, the risk premium effect channel, the Oi-Hartman-Abel effect channel, and real option effect channel, respectively [18]. These four channels have a comparative potential effect, and they may cross each other in a general balanced environment. In terms of the uncertainty affecting the economic activities of "enterprises", the real options theory introduces the idea of “options” into the investment decision of enterprises [19]. At present, the "real options theory" is one of the theories of corporate investment decision-making in an uncertain environment that is widely accepted by the theoretical horizon [20].

Real options theory suggests that the real options effect can cause enterprises to increase (growth options) or decrease (wait and see) their investments [21]. According to the real options theory, the investment opportunities can be regarded as a “call option” held by enterprises [22]. In the presence of the adjustment cost, the enterprise investment means that the enterprise has executed the call option, while the investment cost constitutes the execution price of the option. Therefore, the choice of current investment means that the enterprise gives up the right to wait for better investment opportunities in the future, and this right is valuable to the enterprises. This value is the possible opportunity cost of the current investment [23].

**Fig 1:**
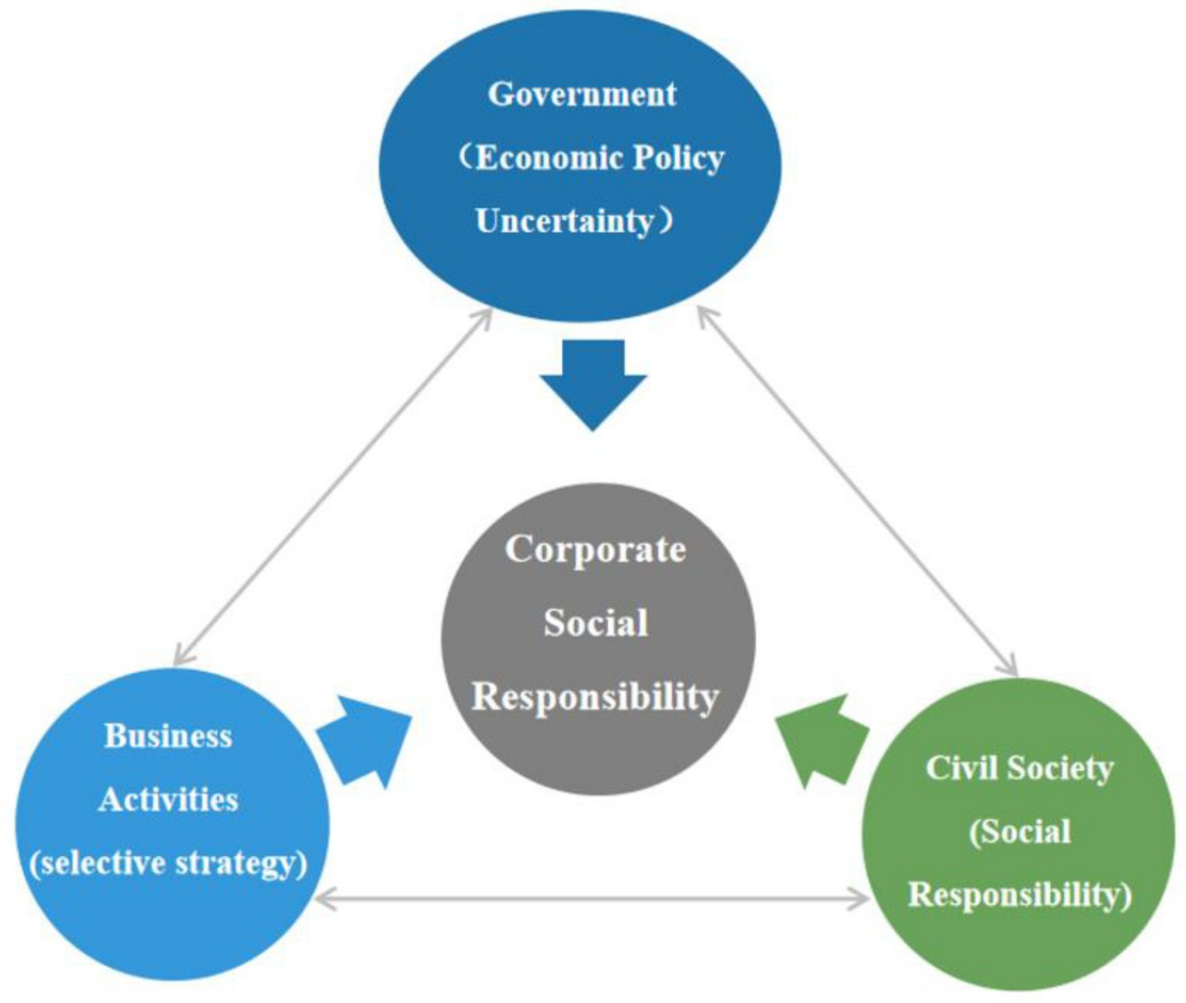
Conceptual mapping of EPU-CSR relationship.

As Gaspar mentioned that, CSR is an investment from which a business should earn tangible returns and have a positive impact on net profits [24]. In other words, the CSR implementation is considered a special long-term asset investment for enterprises [25]. Based on the perspective of enterprise investment, the paper focuses on analyzing the transmission path of economic policy uncertainty affecting corporate social responsibility. Based on the above analysis, the paper presented a conceptual map of government actions to promote and develop corporate social responsibility (Fig 1).

In Fig 1, various types of government economic policies are briefly classified according to different macroeconomic regulation directions. One is social responsibility of government, that is, public policies that the government formulates to improve its own social responsibility and lead by example [26]. The second is CSR in government-enterprises relationships [27], government’s public policies that aim to improve the practices of CSR. Third, CSR in government-society relations [28], government’s public policies that aim to raise awareness among stakeholders in civil society. Fourth is CSR itself; which aims to improve the cooperation among government, enterprise, and society. In Fig 1, economic policy uncertainty has direct or indirect effects on corporate social responsibility through different classifications of policy impacts. There is a clear transmission path, at least conceptually.

For further details, based on the real options theory, the inherent value of the enterprise investment project is decomposed, and the impact of economic policy uncertainty on enterprise investment is specifically reflected in the price of the enterprise products [29]. Using the American perpetual option pricing method to analyze corporate investment behavior [30], the following investment rules were derived [31]. If the economic policy uncertainty is low, so that the sales price of the enterprise’s goods is greater than the investment threshold, the option premium is 0, and the enterprise should choose to invest. If the economic policy uncertainty is high, so that the sales price of goods is less than the investment threshold, the option premium is greater than 0. At this time, it is valuable for enterprises to have investment options, and choose to wait for a better investment opportunity instead of investing immediately, that is, the “wait” effect [32]. It is deduced from this that when economic policy uncertainty is low, firms will increase their CSR investment; when economic policy uncertainty is high, firms will reduce their CSR investment and thus fail to practice CSR. Based on the above theoretical analysis, the research hypothesis on "economic policy uncertainty and corporate social responsibility" is obtained:

***Hypothesis 1 (H1)*:** *Economic policy uncertainty has a negative impact on corporate social responsibility*.

China’s unique economic system determines the relationship between the government and enterprises, enterprises and various sectors, and enterprises with other enterprises, which makes the different institutional and market environment faced by enterprises [33]. On the one hand, the state-owned enterprises assumes more social responsibility in the livelihood of people [34]. In the context of high economic policy uncertainty, state-owned enterprises in the food industry still need to play a leading role in the practice of corporate social responsibility. However, since state-owned enterprises are to some extent controlled by the government [35]. A certain percentage of its decision makers are government officials, and investment decisions exhibit a strong bias toward compliance with economic policy adjustments [36]. Under the high uncertainty of economic policies, the investment decisions in social responsibility cannot wait for opportunities or abandon investments based solely on market conditions [37]. Therefore, the research hypothesis on "corporate social responsibility under different property rights properties" is drawn.

***Hypothesis 2 (H2)*:** *The social responsibility investment behavior of state-owned enterprises is not easily effected by the economic policy uncertainty*.

Enterprises with different market shares have a great asymmetry in the level of development and the market level [38]. On the one hand, the enterprises dominating the market may be exposed to the fluctuating global market [39], which is greatly affected by the uncertainty of macroeconomic policy. That makes enterprises necessary to consider various domestic and foreign macroeconomic factors when making investment decisions [40]. On the other hand, enterprises with high market share have complex capital structure due to their long history of development. When faced with shocks of economic policy uncertainty, the investment behavior of companies in CSR based on the complex capital structure and business strategy will be adjusted accordingly over time [41]. This allows enterprises to minimize economic losses in times of high macroeconomic volatility. Therefore, the research hypothesis on "corporate social responsibility under the difference in market association strength" is drawn.

***Hypothesis 3 (H3)*:** *The practice of corporate social responsibility in strong market-related enterprises is more vulnerable to the economic policy uncertainty*.

## 3 Methodology

### 3.1 Data source

This paper takes the A-shares listed companies from food industry in China as example. The statistical caliber is subject to the “farm & sideline product processing” and “food manufacturing” under the industry classification of the CSRC (China Securities Regulatory Commission), that is referred to as the “food industry” in this paper. To more accurately observe the impact of economic policy uncertainty on corporate social responsibility, the following treatment was conducted based on the initial data. (1) Excluding samples with missing data from financial performance and other variables. (2) Excluding listed companies with special treatment such as “Special Treatment” and “Particular Transfer”. (3) And handling 1% of continuous variables to remove outliers. Due to the existence that some enterprises became A-share listed enterprises from 2009–2018, without artificially eliminating the authenticity of the samples as far as possible, the short panel data of 573 samples was finally obtained.

The data of the CSR index was obtained from the "Hexun Network", and calculated according to the social responsibility report disclosed by the enterprise and the annual report. Hexun.com released a comprehensive social responsibility score of China’s listed companies as a measure of corporate social responsibility performance. Based on the social responsibility reports and financial reports of listed companies in China, the score sets up 13 secondary indicators and 37 tertiary indicators in five aspects: shareholders’ responsibility, employees’ responsibility, suppliers’, customers’ and consumers’ rights and interests’ responsibility, environmental responsibility, and public responsibility. The systematic evaluation of CSR can reflect the social responsibility performance of enterprises in a more comprehensive and objective way. In recent years, it has been increasingly used in related research in China [42].

**Fig 2:**
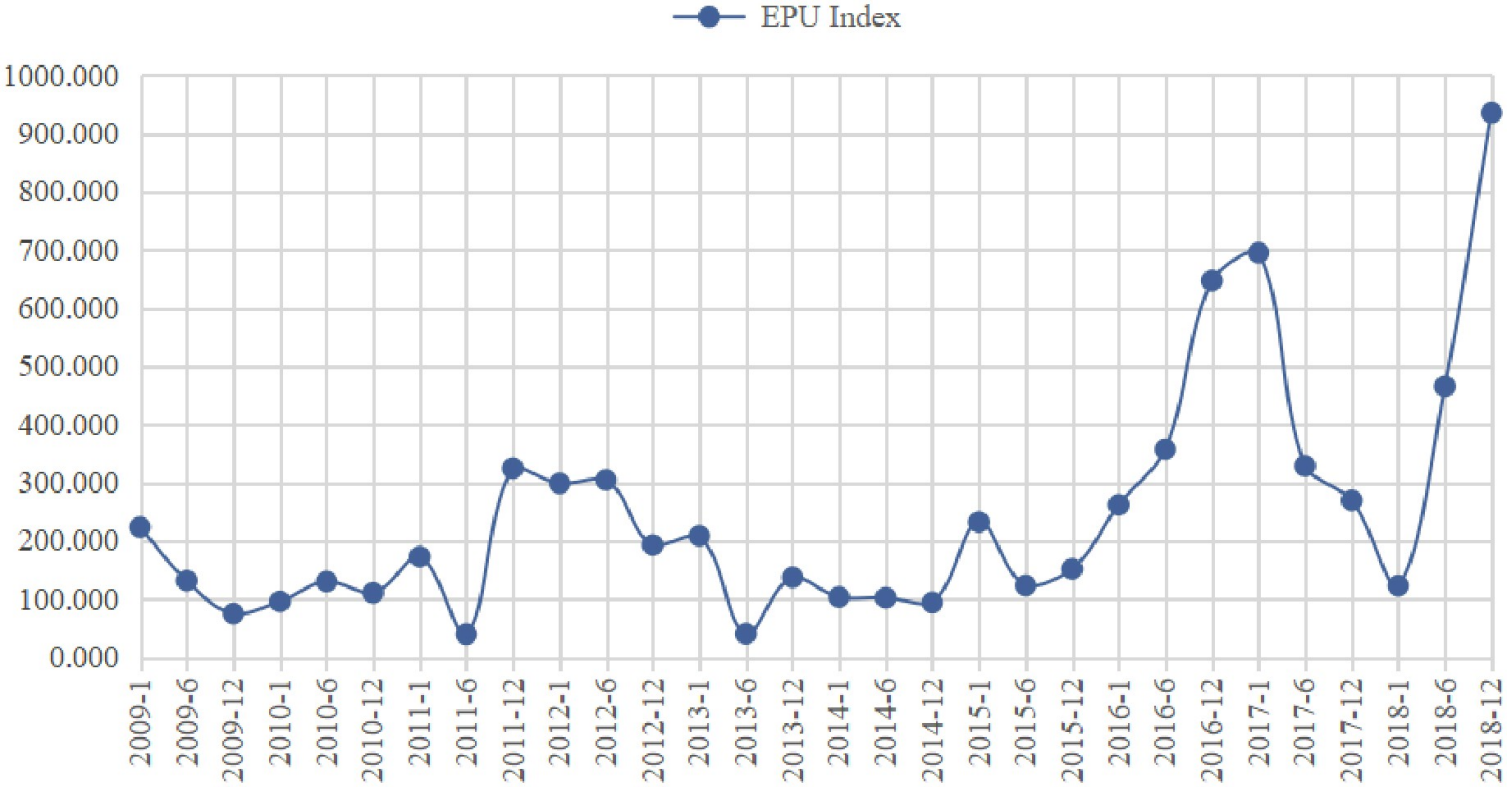
The EPU index of China.

The original financial data of the companies was obtained from the China Stock Market & Accounting Research Database (CSMAR). And the macroeconomic data were obtained from the National Bureau of Statistics. The economic policy uncertainty index is proposed by Baker et al. [43], who speculated on the uncertainty faced by microeconomic subjects based on the concern of the news media. First, large Chinese newspapers are selected, and the reports related to economic policy uncertainty are screened by searching keywords such as uncertainty/uncertain, economic/economy, policy, tax, spending, regulation, and so on. The China EPU index is then obtained after statistical analysis and standardization. Original data from the website of “http://www.policyuncertainty.com/china_epu.html”. Then, this paper selected a monthly index of China’s economic policy uncertainty [44], the descriptive statistics of the economic policy uncertainty index is shown in Fig 2.

In Fig 2, the statistical analysis of China’s EPU index from January 2009-December 2018 reveals that China’s economic policy uncertainty as a whole shows a fluctuating upward trend. Moreover, the economic policy uncertainty index is at a high level in two time periods, December 2011-June 2012 and December 2016-January 2017. This corresponds to a series of major adjustment periods in China government’s macro-regulation, respectively. In 2012 and 2016 were the key points for China’s major economic and political changes [45]. In 2012, the 18th National People’s Congress was held in China, and the change of government at the central and local levels was completed one after another, which brought about changes in macro policies. In 2016, the first year of China’s 13th Five-Year Plan, it was also a crucial year for win a moderately prosperous society in in all aspects. China began to comprehensively carry out supply-side structural reform, and macroeconomic and society have experienced major transformations. This makes the development of all industries of the national economy facing great uncertainties, especially the manufacturing industry [46].

### 3.2 Model specification

This paper focuses on measuring the impact of economic policy uncertainty in the current period on the practice of CSR within the food industry. Following the real options effect channel, the transmission path of economic policy uncertainty (EPU) affecting corporate social responsibility (CSR) is sorted out. Then, the basic panel model is constructed as follows:

$$CSR_{it}=\alpha_{0}+\beta_{1}EPU_{t}+\sum Controls_{it}+\mu_{i}+\varepsilon_{it}$$ (1)

In Eq (1), *CSR*_*it*_ is the corporate social responsibility score in the *t*-year of *i*-enterprise, and *EPU*_*t*_ is the China economic policy uncertainty index in *t*-year. The annual EPU index is obtained by geometric average of the China EPU index. *Controls*_*it*_ for other variables affecting corporate social responsibility [47], including the *Asset*_*it*_ which represens the scale of the company, *Mbr*_*it*_ represents the company growth capacity of operating income growth rate, *Capital*_*it*_ indicates the corporate management of working capital, *Director*_*it*_ shows independent director ratio variables, *Roa*_*it*_ represent the return on assets, *Turnover*_*it*_ indicate total assets turnover, *debt*_*it*_ shows the long-term debt of enterprises. In addition, GDP growth rate is adopted as a time variable to control the macroeconomic situation. The descriptive statistics of all variables is shown in Table 1.

**Table 1:** Descriptive statistics of variables.

Variables	Variables’ meaning	Min	Max	Mean
*CSR*	The score of Corporate social responsibility.	-7.990	90.310	24.508
*YEPU*	The annual index of economic policy uncertainty.	94.643	404.916	227.66
*Growth*	The rate of real GDP after deflating.	0.072	0.164	0.108
*Asset*	Total annual assets of the enterprise.	1.830	427.830	39.176
*Turnover*	Total asset turnover ratio, that is the efficiency of business asset operations.	0.010	5.873	0.777
*Lev*	Gearing ratio, that is total liabilities as a percentage of total assets.	0.025	1.411	0.368
*Roa*	Return on assets, the profitability of total assets.	-0.506	0.577	0.047
*Debt*	Long-term debt, that is debt with a repayment period of more than one year.	0	23.700	1.146
*Capital*	Working capital, that is the net working capital available for business operations.	-28.910	81.980	4.473
*Mbr*	Increase in the rate of main business revenue represents the change in main business income.	-0.993	1.043	0.080
*Director*	Independent director ratio, that is, the number of independent directors as a percentage of the total number of board of directors.	0.200	2.333	0.389

The social responsibility score of A-share listed companies in the food industry from 2009 to 2018 was between -7.990 and 90.310, with an average of 24.508. In 2010–2017, the data on CSR in all industries was fully disclosed. Therefore, by comparing the all industry data from 2010 to 2017, it was concluded that the CSR score was between 29.805 and 43.253, with an average of 39.054. And the average CSR score was increasing year by year. Meanwhile, the average food corporate social responsibility score was only 25.145 during 2010 to 2017. That is, there is a large gap between the CSR evaluation values of food companies and other industries. As a basic industry that related to the livelihood of people, the level of social responsibility of food industry is lower than that of the general industry. To some extent, it reflects the reasons why malignant food safety incidents are still repeatedly banned under the safety supervision and management. Therefore, in the context of the implementation of the food safety strategy, it is very important to explore how to improve the corporate social responsibility of the food industry to ensure China’s food safety.

## 4 Results and analysis

### 4.1 Effect of EPU on CSR

A panel regression model was used to empirically simulate the impact of economic policy uncertainty on the corporate social responsibility of the food industry. The paper reported that the standard errors of the regression model are treated as the robust standard errors of clustering for enterprises. Model 1 and Model 2 are the simulation results of fixed effect model and random effect model, respectively. Through the Hausmann test [48], it was determined to use the fixed effect model (model 1) for analysis.

In addition, a series of robust tests were carried out in this paper. Referring to the relevant research [49], the arithmetic mean of the monthly EPU index of China and the weighted mean of the monthly EPU index were used to replace the geometric mean for regression estimation. In Model 3, the annual EPU index obtained after arithmetic average is included in the model as a variable to measure the economic policy uncertainty. In Model 4, the weighted average of annual EPU calculated with month as the weight was included in the model as a variable to measure the economic policy uncertainty. The basic empirical simulation results and robustness test results are shown in Table 2.

**Table 2:** Results of the base regression.

Variables	Model 1 (fixed effect)	Model 2 (random effect)	Model 3 (robust test 1)	Model 4 (robust test 2)
*YEPU*	-0.013** (0.005)	-0.010* (0.006)	-0.014*** (0.005)	-0.011*** (0.004)
*Growth*	0.022 (0.209)	-0.075 (0.220)	0.010 (0.209)	-0.011 (0.214)
*Asset*	0.047*** (0.018)	0.001 (0.035)	0.048*** (0.018)	0.046*** (0.017)
*Turnover*	0.038*** (0.013)	0.012 (0.012)	0.038*** (0.013)	0.039*** (0.013)
*Lev*	-0.049 (0.047)	-0.038 (0.068)	-0.051 (0.047)	-0.048 (0.047)
*Roa*	0.490*** (0.116)	0.371*** (0.114)	0.489*** (0.115)	0.488*** (0.116)
*Debt*	-0.133 (0.204)	-0.095 (0.232)	-0.119 (0.205)	-0.118 (0.210)
*Capital*	0.157 (0.130)	0.188 (0.152)	0.158 (0.129)	0.155 (0.129)
*Mbr*	-0.014 (0.021)	-0.015 (0.024)	-0.012 (0.021)	-0.011 (0.021)
*Director*	0.049 (0.059)	-0.008 (0.036)	0.053 (0.059)	0.053 (0.059)
*Fixed effect*	Yes	No	Yes	Yes
*Observations*	573	573	573	573
*R-square*	0.2908	0.3898	0.3871	0.3844

Note: The robust standard errors clustered in the firm level in the regression.

*, ** and *** indicate statistical significance at the 10%, 5%, and 1% level respectively.

According to the results of fixed effect simulation of model 1 (as mentioned in Table 2), under the confidence level of 5%, the economic policy uncertainty has a negative impact on corporate social responsibility in the context of the food industry, and the regression coefficient showed a value of -0.013. This result verified hypothesis 1 (H1), as the economic policy uncertainty increases, enterprises in the food industry are less willing to practice in corporate social responsibility. This reveals that the food industry’s "corporate social responsibility" is in line with the real option theory. When the economic policy uncertainty has increased, enterprises are unwilling to bear more social responsibility. In addition, the research results showed that the regression coefficients of total assets, return on assets, and asset turnover on corporate social responsibility are 0.047, 0.490, and 0.038 respectively, which show a significant positive impact. That is, the enterprise’s own operation scale, growth ability, and profit-making ability have a significant impact on the practice of corporate social responsibility. The possible reason is that the larger a company is, the more it spends on CSR for the sake of maintaining a positive corporate image. The higher the asset turnover ratio, the more funds are available to practice CSR, and therefore the higher the CSR score.

According to the robustness test results of Model 3 and Model 4, the impact coefficients of economic policy uncertainty on corporate social responsibility showed values of -0.014 and -0.011, respectively. This finding is consistent with the basic estimates, that is, the basic regression results are stable and reliable.

### 4.2 Results of endogeneity test

There are two main causes of endogeneity, for instance the problem of omitted variables and the explanatory variable with the explained variable are mutually causal, which reveal a reverse causal problem [50]. For the possible omitted variables, the fixed effect of the enterprises was added to the basic regression analysis and the empirical results were subjected to a series of robustness tests. For the possible reverse causality, this paper used the two-stage least square method (2SLS) to verify it. The 2SLS method is a common approach to solving endogeneity problems [51]. This approach is to divide the the endogenous explanatory variables into two parts, with an exogenous part caused by instrumental variables and an endogenous part associated with the disturbance term [52]. In the first stage, the exogenous part of these variables is obtained by predictive regression of the exogenous variables. In the second stage, regression of the explanatory variables on the exogenous part of the explanatory variables to obtain consistent estimates by eliminating bias.

In the endogenous test, India’s economic policy uncertainty was selected as the instrumental variable of China’s economic policy uncertainty [53]. One reason is the similarity between China and India as emerging economies; the other is the increasingly close trade relationship and mutual influence between China and India [54]. And the economic policy uncertainty in one country can hardly directly determine the practice of corporate in other countries [55]. The principles of relevance and exclusivity of instrumental variables are satisfied at the same time [56]. Therefore, this paper selected India’s EPU index as the instrumental variable of the China’s economic policy uncertainty. And after the weak instrumental variable test, the statistics values of *Cragg-Donald Wald F* were greater than the empirical value of 10, indicating that the choice of instrumental variables is reliable.

Taking A-shares listed companies in the food industry as an example, the 2SLS method was used to analyze whether economic policy uncertainty is endogenous to corporate social responsibility (results are shown in Table 3). According to the regression results of the second stage, the coefficient of economic policy uncertainty on corporate social responsibility showed a value of -0.071. The regression results showed consistency with the basic estimates, and the endogenous problems were basically excluded.

**Table 3:** Regression results for instrumental variables.

Variables	First stage			Second stage		
	Coefficient	Robust Standard Error	*t-value*	Coefficient	Robust Standard Error	*t-value*
*YEPU*	——	——	——	-0.071***	0.016	-4.41
*IEPU*	-1.566***	0.160	-9.80	——	——	——
*Growth*	1.131	1.711	0.66	-0.641**	0.306	-2.09
*Asset*	0.204***	0.079	2.59	0.075***	0.018	4.12
*Turnover*	-0.121*	0.063	-1.93	0.026*	0.015	1.76
*Lev*	-0.749***	0.236	-3.18	-0.095**	0.049	-1.94
*Roa*	0.151	0.633	0.24	0.679***	0.175	3.87
*Debt*	1.567	1.494	1.05	-0.085	0.286	-0.30
*Capital*	0.098	0.492	0.20	0.151	0.096	1.58
*Mbr*	0.123	0.155	0.79	0.007	0.025	0.30
*Director*	0.610***	0.228	2.68	0.184**	0.084	2.19

Note:

*, ** and *** indicate statistical significance at the 10%, 5%, and 1% level respectively.

### 4.3 Heterogeneous effects of different enterprises

As mentioned above, the government drives enterprises to practice social responsibility, and the nature of property rights of enterprises affects their anti-risk ability to deal with policy uncertainty. Additionally, the market share of enterprises affects the ability of companies to practice corporate social responsibility under economic fluctuations. In this regard, grouped regressions were conducted to observe the differential impact of economic policy uncertainty on CSR in different groups. The subgroups are the state-owned enterprises group compared to the nonstate-owned enterprises group, and the strong market-related enterprises group compared to the weak market-related enterprises. Table 4 indicates the comparison of the group regression results.

**Table 4:** Results of the group regression.

Variables	State-owned enterprise	Non-state-owned enterprises	Strong market correlation enterprises	Weak market correlation enterprises
*YEPU*	0.003 (0.007)	-0.021*** (0.007)	-0.022** (0.009)	-0.003 (0.005)
*Growth*	0.225 (0.276)	-0.071 (0.277)	-0.390 (0.324)	0.252 (0.241)
*Asset*	0.037** (0.019)	0.072*** (0.024)	0.044** (0.022)	0.086 (0.154)
*Turnover*	0.029 (0.037)	0.045*** (0.013)	0.043*** (0.014)	0.007 (0.022)
*Lev*	-0.116 (0.114)	-0.042 (0.058)	-0.109 (0.100)	-0.073* (0.044)
*Roa*	0.421* (0.251)	0.460*** (0.138)	0.664*** (0.205)	0.398*** (0.149)
*Debt*	-0.319 (0.673)	-0.256 (0.244)	-0.194 (0.238)	0.602 (1.230)
*Capital*	0.037 (0.136)	0.158 (0.204)	0.041 (0.132)	0.203 (0.223)
*Mbr*	-0.025 (0.035)	-0.003 (0.029)	-0.029 (0.027)	-0.001 (0.027)
*Director*	0.013 (0.064)	-0.051 (0.200)	0.061 (0.081)	0.183 (0.118)
*Fixed effect*	Yes	Yes	Yes	Yes
*Observations*	207	366	280	293
*R-squared*	0.3017	0.4414	0.4282	0.2502

Note:

*, ** and *** indicate statistical significance at the 10%, 5%, and 1% level respectively. Robust standard errors are in parentheses.

In Table 4, the impact coefficient of economic policy uncertainty on corporate social responsibility in the sample of nonstate-owned enterprises was estimated at a value of -0.021, which reveals that hypothesis 2 (H2) can be accepted. That is, the state-owned enterprises in the food industry are not vulnerable to the uncertainty of economic policies in practicing social responsibility, while the performance of corporate social responsibility of non-state-owned enterprises depends on macroeconomic policies. The possible reason is that, compared with nonstate-owned enterprises, state-owned enterprises take more corporate social responsibilities in addition to profit-making purposes, especially for the food industry. State-owned enterprises are basically related to the national economy and people’s livelihood. Secondly, due to the existence of institutional correlation, to a certain extent, state-owned enterprises are easier to obtain economic policy related information than non-state-owned enterprises, and these enterpriese are less affected by policy fluctuations.

The market relevance of enterprises is judged by the difference between the total assets of the enterprises and the industry average value. The higher the total assets of the enterprises, the higher the market share of the enterprises and the fact that they belong to a group with a strong market relevance [57]. Therefore, enterprises with total assets are greater than the industry average value, and are divided into the group of strong market correlation. According to the results of the group regression, the regression coefficient of the strong market correlation group showed a value of -0.022, which is significant at the 5% confidence level and therefore, the hypothesis 3 (H3) can be accepted. That is, the social responsibility performance of strong market related enterprises is more vulnerable to the uncertainty of economic policy than that of the weak market correlation group. The results further showed that the expansion of the enterprise scale has enhanced the ability of enterprises to resist risks and created the necessary material conditions to practice in social responsibility. However, at the same time, the problems caused by the expansion of enterprise scale are more serious when faced with a complex market environment. Large enterprises incorporate several production and operation units into the same organizational system, so that enterprises have less flexibility to adjust their strategies when facing the unpredictable macroeconomic uncertainty risks. Which indicates that, enterprises need to consider many factors in market investment decisions, such as social responsibility.

## 5 Discussion

Previous studies have confirmed that the external issue of economic policy uncertainty has a significant impact on the internal activities of enterprises [12]. For example, Li et al. took the A-share listed companies from the 2002 to 2013 as example [58], and empirical research found that economic policy uncertainty has an inhibitory effect on corporate investment. However, corporate social responsibility is a special corporate investment behavior [24]. For enterprises, the implementation of corporate social responsibility is related to sustainable development. For the government and civil society, the implementation of corporate social responsibility is related to social well-being, especially for food companies [7]. Therefore, this paper considers the impact of economic policy uncertainty on the social responsibility of food companies.

In comparison with the previous study [26], this study reports that economic policy uncertainty has a negative impact on corporate social responsibility through empirical research. This also verifies the research conclusion that macroeconomic uncertainty has an inhibitory effect on corporate investment behavior, from the perspective of corporate socially responsible. In addition, Wang et al. [59] took A-share listed companies from 2008 to 2017 as an example, to analyze the impact of economic policy uncertainty on disclosure of corporate social responsibility information. The results show that when the EPU is aggravated, the corporate willingness to release a social responsibility report is significantly enhanced, including the information disclosure quality, but the expected future social responsibility rating gradually declines. On this basis, this paper validates the prediction that CSR scores decline when economic policy uncertainty increases.

Unlike previous studies, some studies believe that macroeconomic uncertainty has a more prominent impact on the internal business activities of state-owned enterprises [31]. For example, Zhao et al. [60] taking China’s capital market as an example, argued that the inhibitory effect of economic policy uncertainty on CSR-related behavior is more pronounced in state-owned enterprises. However, this study takes listed companies in China’s food industry as the research object, and the study finds that economic policy uncertainty has a more prominent inhibitory effect on the corporate social responsibility of nonstate-owned enterprises. It may be caused by the particularity of food enterprises. Compared to other types of enterprises, government regulation and social supervision are more intense for food enterprises to practice social responsibility, especially for state-owned enterprises. Therefore, regardless of whether they face greater economic uncertainty, state-owned enterprises that produce or process food are more likely to practice social responsibility for government image or political purposes. However, for nonstate enterprises, they are more likely to practice CSR voluntarily. Due to the current noncompulsory nature of CSR practices, when economic policy uncertainty is heightened, it may be motivated by economic benefits and thus reduced the fulfillment of CSR.

## 6 Conclusion and implications

This paper utilized data of A-share listed companies in China’s food industry from 2009 to 2018 and matched panel data of 86 A-share listed companies. This paper further established the transmission relationship between macroeconomic policy uncertainty and microcorporate social responsibility and empirically analyzed the choice of social responsibility of food enterprises under economic policy uncertainty. The results showed that economic policy uncertainty will significantly reduce the performance of enterprises in social responsibility. The basic research conclusion revealed the fact that in the period of high economic policy uncertainty, enterprises adopt a "watching and waiting" attitude to reduce their investment in social responsibilities. Therefore, this negative attitude is likely to have an adverse impact on food safety of consumers and national trade. Further, from the perspective of property rights and market relevance, this paper analyzed the choice differences of food enterprises when faced with economic policy uncertainty. In the high economic uncertainty environment, non-state-owned enterprises with asymmetric information and enterprises with large market share are more vulnerable in the practice of social responsibility. Based on the above analysis, the following policy implications are suggested:

1. The government should set up a special fund for the development of the food industry. When macroeconomic fluctuations are raised, enterprises in the food industry are generally conservative in their investment social responsibility. The government should appropriately provide special funds to support and subsidize the implementation of corporate social responsibility, especially for small and medium-sized enterprises in the food industry. The government should properly support enterprises to deal with the risk of policy uncertainty, in order to promote enterprises in the food industry to better practice social responsibility. These enterprises are responsible for consumers at home and abroad, society, environment, and ensure China’s food safety.
2. The government should formulate national standards for the social responsibility of the food industry. Compared to other industries, the food industry is more closely related to livelihood of people. There is not only need to have standard guidelines, but also need to have strict unified standards to restrain enterprises, especially nonstate enterprises operating behavior. Therefore, enterprises can take social responsibility as much as possible in the process of producing food and selling products, and ensure that the food industry can better practice corporate social responsibility with strict industry standards.
3. The government should strengthen the supervision of social responsibility of food enterprises. Enterprises with a higher market share pay more attention to the social image of enterprises. In view of the development status of corporate social responsibility in the initial stage, it is suggested that the social responsibility report of food enterprises should be made public, so that the enterprises can be subject to public supervision on the basis of government supervision. Only when enterprises practice social responsibility openly and transparently, and the public has the right to know, then the frequent occurrence of public events related to food safety can be reduced.

In light of the above findings, it is important to note the limitations of this study. As there is no unified measurement standard of economic policy uncertainty, this paper selected widely China’s economic policy uncertainty index more objective and as possible. In future research, it is necessary to optimize the theoretical model under different measurement methods with respect to the impact of macroeconomic policy uncertainty on microeconomic fluctuations. Further studies are required to minimize the simulation errors and improve the reference for the study of micro effects of macroeconomic uncertainties.
